# Recurrent gastric volvulus associated with a gastrointestinal stromal tumor: A case report

**DOI:** 10.1016/j.ijscr.2019.03.031

**Published:** 2019-03-30

**Authors:** Ryohei Murata, Yo Kamiizumi, Chihiro Ishizuka, Sayuri Kashiwakura, Takeshi Tsuji, Hironori Kasai, Yasuhiro Tani, Tsutomu Haneda, Tadashi Yoshida, Kenzo Okamoto, Koji Ito

**Affiliations:** a Department of Surgery, Iwamizawa Municipal Hospital, 068-8555, Iwamizawa-shi, Japan; b Department of Gastroenterological Surgery I, Hokkaido University Graduate School of Medicine, 060-8648, Sapporo-shi, Japan; c Department of Pathology, Hokkaido Chuo Rosai Hospital, 068-0004, Iwamizawa-shi, Japan

**Keywords:** GV, gastric volvulus, GIST, gastrointestinal stromal tumor, SMT, submucosal tumor, CT, computed tomography, Gastrointestinal stromal tumor, Submucosal tumor, Gastric volvulus, Gastrectomy, Acute abdominal syndrome, Epigastric pain

## Abstract

**Introduction:**

Gastric volvulus (GV) is defined as a rotation of the stomach along its short or long axis leading to variable degrees of gastric outlet obstruction. Rotation of the stomach >180° may cause closed loop obstruction and possible strangulation, which often causes acute abdominal pain. Strangulation and gangrene of the twisted stomach sometimes occurs, which demands immediate surgical intervention. We report a case of acute gastric volvulus due to a gastrointestinal stromal tumor (GIST), with multiple recurrences, that eventually required emergency gastrectomy.

**Presentation of the case:**

A 71-year-old woman with a history of recurrent epigastric pain, nausea, and anorexia was diagnosed to have a 70-mm sized submucosal tumor (SMT) in the lesser curvature of the stomach. An elective gastrectomy was planned; however, before the procedure, she visited the emergency room with acute recurrent epigastric pain associated with postural variations. Computed tomography (CT) revealed a GV and the tumor had shifted to the greater curvature. An emergency gastrectomy was performed. The postoperative course was uneventful and pathological examination revealed features consistent with that of GIST.

**Discussion:**

GV with GIST has rarely been reported and risk factors such as size or localization are unknown. In this case, GV was probably caused by GIST of the stomach, which was large and heavy enough to rotate the gastric body around the mesenteroaxis.

**Conclusion:**

Surgical intervention without delay should be planned in similar scenarios accounting for the risk of GV in GIST.

## Highlights

• A 7-cm gastrointestinal stromal tumor (GIST) caused recurrent abdominal pain, apparently through gastric volvulus (GV).
• The risk of GV based on the size and location of a tumor is unknown.
• Prompt surgical intervention is indicated in cases of recurrent GV.

## 1 Introduction

Gastric volvulus (GV) is defined as a rotation of the stomach along its short or long axis leading to variable degrees of gastric outlet obstruction. Rotation of the stomach >180° may cause closed loop obstruction and possible strangulation which often causes acute abdominal pain. Strangulation and gangrene of the twisted stomach sometimes occurs, which demands immediate surgical intervention [1]. We report a case of acute gastric volvulus due to a gastrointestinal stromal tumor (GIST), with multiple recurrences, that eventually required emergency gastrectomy. This case has been reported in line with the SCARE criteria [2].

## 2 Presentation of case

**Fig. 1:**
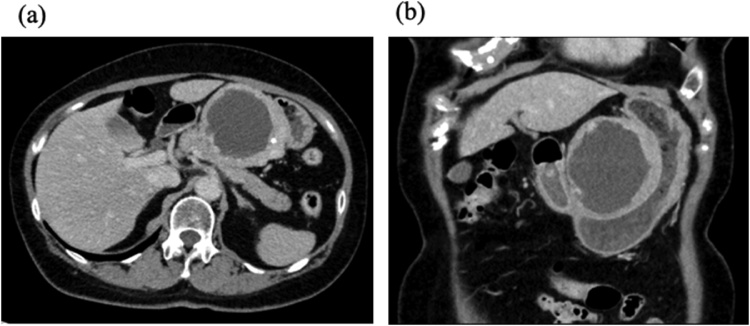
Abdominal enhanced computed tomography. **a/b.** A smooth-surfaced tumor measuring 70 × 70 mm is noted at the lesser curvature of the middle stomach. The tumor has an internal low-density area and a thick peripheral high intensity area which has an unclear boundary between the stomach. The tumor was suspected to be a submucosal tumor.

A 71-year-old woman, who had previously been diagnosed with a 45-mm sized tumor a year ago, visited her local primary doctor with complaints of recurrent episodes of epigastric pain, nausea, and anorexia. On upper gastric endoscopy, a 70-mm submucosal tumor (SMT) was found at the gastric angle (Fig. 1a and b). The tumor was observed at the lesser curvature of the middle stomach, with an internal low-density area and a thick peripheral high intensity area which had an unclear boundary between the stomach. The tumor was suspected to be a submucosal tumor.

**Fig. 2:**
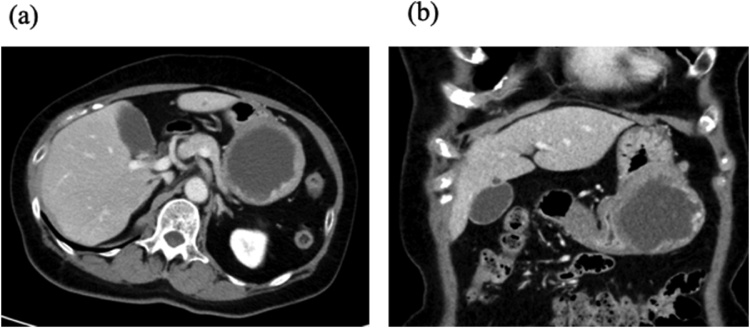
Enhanced abdominal computed tomography at the onset of abdominal pain. **a/b.** The gastric tumor had moved to the side of the greater curvature and the stomach was twisted. There is no evident contrast failure area of the stomach.

She had a past medical history of hypertension, herpes zoster, delivery by cesarean section, gastritis, and cholelithiasis. We had planned to perform an elective gastrectomy, but while awaiting the surgery, she came to our hospital with complaints of sudden abdominal pain. A computed tomography (CT) showed that her stomach had twisted organoaxially and the SMT had moved to the side of greater curvature (Fig. 2a and b).

**Fig. 3:**
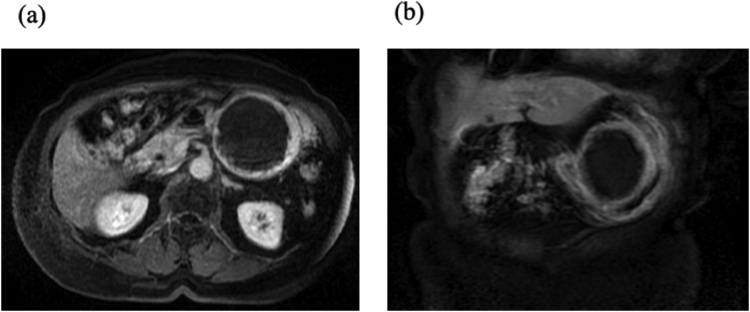
Gadolinium-enhanced abdominal MRI. **a/b.** There is a smooth-surfaced tumor of 70 × 70 mm at the lesser curvature of the middle stomach and almost all parts of the tumor had a high intensity on T2WI. The submucosal tumor had relocated to the original position.

Laboratory tests were within the normal ranges. She was admitted to our hospital and kept in the right lateral decubitus position, which relieved her pain. Abdominal magnetic resonance imaging (MRI) on the following day, which had been already planned to examine the characteristics of the tumor, showed that the tumor had returned to its original position (Fig. 3a and b).

**Fig. 4:**
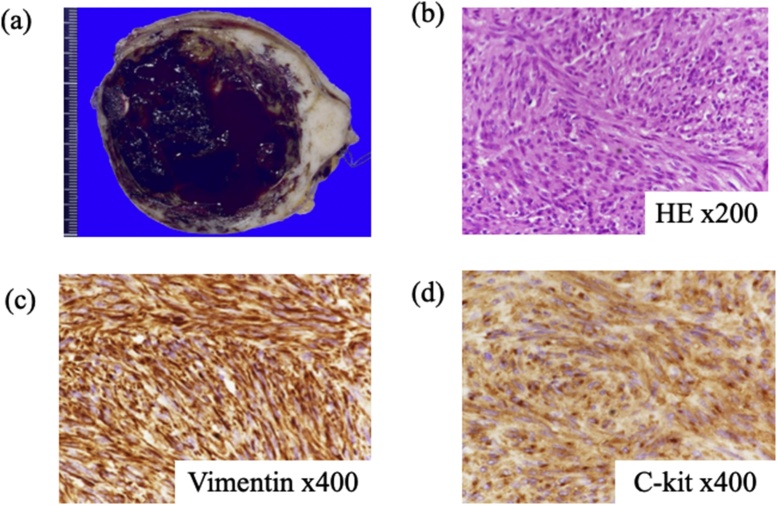
Histopathological examination of the first tumor specimen. **a.** The cut surface of the white tumor. The tumor had a smooth surface and enclosed a cystic cavity containing a blood clot. **b.** Hematoxylin and eosin staining of the specimen showed long spindle-shaped cells lined in a bundle with a randomized complex arrangement and growth. **c/d.** Immunostaining with C-kit, CD34 was positive.

During the course of rescheduling the elective gastrectomy, her abdominal pain and nausea recurred throughout the day, especially when she changed her body posture. We attributed the recurrent pain to the occurrence of recurrent GV and decided to perform an emergent gastrectomy on the same day. Via a median incision, we found a tumor at the lesser curvature of stomach, which was resected along a line 2 cm away from the tumor with an electric scalpel and a harmonic scalpel and was sutured with an absorbable thread in the direction of the long axis. The operation time was 137 min and the blood loss was 104 ml. The tumor size was 90 × 73 mm and the weight was 224.5 g (Fig. 4a).

The patient had an uneventful recovery and was discharged on the 14th postoperative day. The histopathological findings of the tumor specimen showed long spindle-shaped cells lined in a bundle with randomized complex arrangement and growth, in a storiform pattern (Fig. 4b). There were some swollen and enlarged cells, with moderate cellular atypia and density. The tumor cells were immunohistochemically positive for C-kit, CD34, DOG-1, and vimentin, but was devoid of SMA, desmin, and S-100. The number of mitotic cells was 2 on a 50 high-power field (HPF) (Fig. 4c and d) We diagnosed a GIST with intermediate risk. The patient comes to our hospital for ambulant follow-up and she has had no recurrence until now.

## 3 Discussion

GV occurs due to torsion of the stomach beyond its physiological range and causes obstruction to the passage of food [1]. The pattern of volvulus is categorized into two types, organoaxial (long axis) and mesenteroaxial (short axis). The organoaxial volvulus pattern comprises nearly 60% of all cases. There are 2 types of GV: one is idiopathic with no specific cause and the other is secondary following esophageal hiatal hernia, diaphragmatic relaxation or elevation, or wandering spleen, among others [3]. GV is more common in infants which is often idiopathic due to the immature stomach and ligaments of the stomach, but cases in adults, often due to secondary causes, have increased recently [4].

Although some cases of GV with gastric cancer have been reported, GV with GIST have rarely been reported. We searched PubMed using the keywords “gastric volvulus” and “gastrointestinal stromal tumor.” Only one case was reported and the risk of GV based on the tumor size and position was unknown [5]. In our case, the GIST was large and heavy enough to rotate the gastric body around the mesenteroaxis, and the attacks of pain recurred with every change in posture. We attributed the pain to the rotation and strangulation. There are acute onset and chronic scenarios; chronic GV presents with mild symptoms and may be diagnosed by radiographic examination in most cases. The triad of symptoms in acute GV is known as Borchardt’s triad, which includes epigastric abdominal distention, fruitless attempts at vomiting, and inability to pass a nasogastric tube [6]. Although the symptoms are present in 70% of patients with GV, a GV cannot be diagnosed by only the symptoms and physical examination. CT imaging and barium meals are recommended to diagnose GV. In this case, both the diagnosis and recovery of GV were made using CT and MRI [6].

Tanner suggested methods of surgical repair for GV. These included diaphragmatic hernia repair, simple gastrectomy, gastropexy and gastrocolic ligament repair (Tanner’s procedure), gastrectomy, gastrojejunostomy, fundoantral gastrostomy (Opolzer’s procedure), and repair of the diaphragm [4].

Insertion of a nasogastric tube and endoscopic reduction are sometimes effective as GV treatment, especially for the mesenteroaxial type which has no closure of the cardiac orifice [1]. Percutaneous endoscopic gastropexy is also useful to prevent recurrence of GV [7]. Even if the torsion was corrected, 30% of GV cases experience recurrence within 26 months; therefore, surgical intervention to prevent recurrence is necessary [8]. In our case, GV was considered to be due to the weight of the GIST and the GIST itself was bigger than 5 cm, which is the size at which surgical resection is recommended [9]. Hence, we performed a gastrectomy as a treatment for the GIST itself and also for GV. There was no recurrence of GV and GIST at 6 months after the surgery.

## 4 Conclusion

A GIST might cause GV and surgical intervention should be considered to prevent GV. Recurrence of GV can occur; therefore, immediate surgical intervention should be planned as soon as practicable if the case has a surgical indication oncologically.

## Conflict of interest

None of the authors has any conflict of interest to declare.

## Funding

This report did not receive any specific grant from funding agencies in the public, commercial, or not-for-profit sectors.

## Ethical approval

Ethical approval has been exempted by our institution.

## Consent

Written informed consent was obtained from the patient for the publication of this case report and accompanying images. A copy of the written consent is available and can be reproduced whenever needed.

## Author contribution

RM performed the operation and perioperative management of the patient. RM also acquired and interpreted the data and drafted the manuscript. YK, CI, SK, TT, HK, YT, TH, TY, and KI participated in the operation, perioperative management of the patient, and revision of the manuscript. KO reviewed pathological findings. All authors read and approved the final manuscript.

## Registration of research studies

Not applicable.

## Guarantor

Ryohei Murata.

## Provenance and peer review

Not commissioned, externally peer-reviewed.
